# *Cinnamomum zeylanicum* bark essential oil induces cell wall remodelling and spindle defects in *Candida albicans*

**DOI:** 10.1186/s40694-018-0046-5

**Published:** 2018-02-09

**Authors:** Zinnat Shahina, Amira M. El-Ganiny, Jessica Minion, Malcolm Whiteway, Taranum Sultana, Tanya E. S. Dahms

**Affiliations:** 1 Department of Chemistry and Biochemistry, University of Regina, 3737 Wascana Parkway, Regina, SK Canada; 2 Microbiology and Immunology Department, Faculty of Pharmacy, Zagazig University, Zagazig, Egypt; 3 Regina Qu’Appelle Health Region, Regina, SK Canada; 4 Centre for Structural and Functional Genomics, Concordia University, Montreal, QC Canada

**Keywords:** *Cinnamomum zeylanicum*, *Candida albicans*, Essential oil, Cell wall remodelling, Spindle defects

## Abstract

**Background:**

Cinnamon (*Cinnamomum zeylanicum)* bark extract exhibits potent inhibitory activity against *Candida albicans* but the antifungal mechanisms of this essential oil remain largely unexplored.

**Results:**

We analyzed the impact of cinnamon bark oil on *C. albicans* RSY150, and clinical strains isolated from patients with candidemia and candidiasis. The viability of RSY150 was significantly compromised in a dose dependent manner when exposed to cinnamon bark oil, with extensive cell surface remodelling at sub inhibitory levels (62.5 μg/mL). Atomic force microscopy revealed cell surface exfoliation, altered ultrastructure and reduced cell wall integrity for both RSY150 and clinical isolates exposed to cinnamon bark oil. Cell wall damage induced by cinnamon bark oil was confirmed by exposure to stressors and the sensitivity of cell wall mutants involved in cell wall organization, biogenesis, and morphogenesis. The essential oil triggered cell cycle arrest by disrupting beta tubulin distribution, which led to mitotic spindle defects, ultimately compromising the cell membrane and allowing leakage of cellular components. The multiple targets of cinnamon bark oil can be attributed to its components, including cinnamaldehyde (74%), and minor components (< 6%) such as linalool (3.9%), cinamyl acetate (3.8%), α-caryophyllene (5.3%) and limonene (2%). Complete inhibition of the mitotic spindle assembly was observed in *C. albicans* treated with cinnamaldehyde at MIC (112 μg/mL).

**Conclusions:**

Since cinnamaldehyde disrupts both the cell wall and tubulin polymerization, it may serve as an effective antifungal, either by chemical modification to improve its specificity and efficacy or in combination with other antifungal drugs.

**Electronic supplementary material:**

The online version of this article (10.1186/s40694-018-0046-5) contains supplementary material, which is available to authorized users.

## Background

*Candida albicans*, a commensal fungi, develops into a resilient pathogen under low host immunity such as that for immunocompromised individuals with HIV/AIDS, and patients undergoing cancer chemotherapy [1–3]. A limited number of antifungals are available for treating such infections, and the use of these antifungal classes, including azoles, echinocandins, polyenes and allylamines, can be accompanied by side effects. Poor bioavailability requires higher doses, which can ultimately result in resistance and ineffectiveness [4]. The continued high morbidity following systemic fungal infection and emerging resistance to antifungal agents underscore a clear need for alternatives [5]. In this context, essential oils (EOs) are gaining popularity due to their strong antimicrobial and antibiofilm activity [6, 7]. EO combinations with other essential oils or existing antifungal agents could vastly reduce the probability of multi-drug resistance [6–11]. However, comprehensive studies are required to fully assess their independent pharmacological properties and potential side effects prior to consideration for clinical use as antifungal agents.

Cinnamon oil is an aromatic liquid obtained from the twigs, bark and leaves of *Cinnamomum zeylanicum* [12]. Extracts of cinnamon bark (CNB) and leaves (CNL) have been used extensively as therapeutics in many cultures since antiquity. The anti-candida activity of CNB oil against planktonic and biofilm culture of *C. albicans* and *non*-*albicans* spp. has been documented [7, 13–15]. The main constituents of CNB oil include trans-cinnamaldehyde, and minor components such as eugenyl acetate, linalool, and benzyl benzoate, each having antifungal activity [16–20]. CNB oil has been shown to alter cell membrane permeability and fluidity, and inhibit biofilm formation [7, 13, 15, 21], but the mechanisms of toxicity remain unknown. On the other hand, each component has been extensively studied, showing effects at various cellular sites, including the cell membrane and cytosol. For example, cinnamaldehyde, the major constituent of CNB oil, targets the membrane and causes increased cell wall thickness in *C. albicans* [16], attributed to β-1-3-glucan synthase inhibition as observed in *Saccharomyces cerevisiae* [22]. The increase in bud scar formation upon cinnamaldehyde exposure also suggests an impact on cell division, resulting in decreased viability [16, 23]. Benzyl benzoate and linalool affect membrane fluidity and induce cell cycle arrest at the G2-M and G1 phases, respectively [20] at concentrations greater than the minimum inhibitory concentration (MIC) [7, 16, 17, 23]. We hypothesized that the cell wall and membrane are primary targets of CNB oil, which in turn disrupt intracellular processes vital to *Candida* survival.

Here, we report a detailed characterization of the anticandidal effects of CNB oil using atomic force microscopy (AFM), laser scanning confocal microscopy (LSCM) and traditional biochemical assays. AFM quantitative imaging (QI™) is a powerful tool for assessing the impact of antifungals [24–28], nutrient stress [29], oxidative stress [30] and characterizing yeast genetic mutants [31], while LSCM imaging of fluorescent markers can delineate defects in intracellular processes. AFM was used to quantify the morphological, ultrastructural and biophysical properties of RSY150 and a clinical isolate exposed to CNB oil. The RSY150 strain of *C. albicans* with RFP tagged histone protein B (Htb-RFP) and GFP tagged β-tubulin (Tub2-GFP) was used to track cell cycle defects in response to CNB oil exposure. Finally biochemical assays were used to verify physiological changes identified by imaging. We report for the first time that CNB oil causes β-tubulin depolymerisation and cell cycle arrest, which we attribute to its major constituent cinnamaldehyde.

## Methods

### Chemicals and media

The cinnamon bark essential oil (Chemical Abstract Service (CAS), registry number 8015-91-6) was a steam distilled extract from the dried inner bark (Now foods, USA) of *Cinnamon zeylanicum* (cinnamon). Bacto™ agar, yeast extract and peptone were obtained from Difco (BD Biosciences, NJ, USA), and all other chemicals were purchased from Sigma Chemical Co. (St. Louis, MO, USA).

### CNB essential oil analysis

CNB oil was analyzed using gas chromatography-flame ionization detection (GC-FID) and gas chromatography–mass spectrometry (GC–MS) with an Agilent 7890A GC according to previously reported methods [32, 33]. Briefly, 1 μl of diluted CNB oil (1:10 in ethanol) was injected onto a HP-5 column (30 mm × 0.32 mm × 0.25 µm) and separated using a carrier gas of helium (constant flow of 1.1 mL/min) with the following temperature gradient: 60 °C for 5 min, increased to 210 °C at 3 °C/min and to 260 °C at 10 °C/min. The GC-FID injector and detector temperatures were 250 °C and the split ratio of injection was 20:1. For GC–MS using similar conditions, 0.2 μl was injected with a split ratio of 200:1 and separated on a HP-5MS column (30 mm × 0.25 mm × 0.25 um).

Retention indices (RI) were determined using a C8-20 standard (~ 40 mg/L each, in hexanes) for both the GC-FID and GC–MS methods. RI values were averaged between the methods for each component of the oil and identified using the NIST14 database and reported literature [32, 34].

### Strains and culture conditions

*Candida albicans* strains and clinical isolates used in this study are described in Table 1. The RSY150 and RSY35 strains were a kind gift of Dr. Richard J. Bennett. RSY150 expresses Tub2-GFP and Htb-RFP and RSY35 is a Kar3 deletion mutant that express only Tub2-GFP [35]. Kar3 is a bifunctional protein having a kinesin-like motor domain joined to a distinct microtubule binding domain that is essential for yeast nuclear fusion during mating [36]. Strains were stored as 50% glycerol stocks at − 80 °C and were freshly revived on yeast-extract peptone dextrose agar (YPDA) containing 1% Bacto-yeast extract, 2% Bacto-peptone, 2% glucose and 2% Bacto-agar prior to each experiment. All strains were grown with continuous shaking (200 rpm) at 30 °C in YPD broth and the mutant strains supplemented with 80 mg/L uridine (YPDU). For the yeast to hyphal transition and leakage assays, cells were grown to mid log phase before exposure to CNB oil. For hyphal induction experiments, cells were grown in YPD broth with 10% fetal bovine serum and 2% glucose.

**Table 1:** *Candida albicans* strains used in this study

Strain	Genotype	References
RSY150	*TUB2*-*GFP*-*SAT1/TUB2*_ *HTB1*-*RFP*-*ARG4*_*/HTB1*_ *arg4*_/_	[35]
RSY35	*leu2::hisG/leu2::hisG his1::hisG/his1::hisG arg4::hisG/arg4::hisG kar3::LEU2/kar3::HIS1 TUB2/TUB2*-*GFP::SAT1*	[35]
CASS1	*his3::hisG/his3::hisGleu2::tetRGAL4AD*-*URA3/LEU2*	[37]
ATCC 64548	Reference strain	Cedarlane Labs, Ontario, Canada
ATCC 10231	Reference strain for clinical isolates	RQHR, Regina,
SK, Canada		
*C. albicans* (1-4)	Clinical isolates; 2 blood, 2 genital	RQHR, Regina, SK, Canada
	*C. albicans* knockout mutants	
VPS28	*his3::hisG/his3::hisGleu2::tetRGAL4AD*-*URA3/LEU2*-*VPS28*	[37]
CRH11	*his3::hisG/his3::hisGleu2::tetRGAL4AD*-*URA3/LEU2*-*Crh11*	[37]
SSU81	*his3::hisG/his3::hisGleu2::tetRGAL4AD*-*URA3/LEU2*-*SSU81*	[37]
DFG5	*his3::hisG/his3::hisGleu2::tetRGAL4AD*-*URA3/LEU2*-*DGF5*	[37]

### Minimum inhibitory concentration (MIC)

The MIC of CNB oil was determined for all strains listed in Table 1 following the guidelines of the Clinical and Laboratory Standards Institute [CLSI 2014] [38] and previously reported method [15], with slight modifications. Briefly, 100 µl of CNB oil (stock concentration 1000 µg/mL) was serially diluted in triplicate in the wells of flat-bottom polystyrene 96-well microtiter plates (Sarstedt, Nümbrecht, Germany). A suspension (OD_600_ = 0.001) of *C. albicans* (2.2 × 10^5^ cells/mL) in YPD was added, with appropriate positive (amphotericin B) and negative (*Candida* only in media) controls and a blank (CNB oil in media) included. The microtiter plates were sealed with parafilm prior to incubation at 30 °C to avoid oil evaporation, and the OD_600_ recorded after 24 h (Biotek Epoch; Northern Vermont, USA). The endpoint was defined as the lowest concentration of the compound resulting in total inhibition (MIC 100%) of growth, compared to the growth in negative control wells. All experiments were performed in triplicate. The MICs for cinnamaldehyde (stock concentration 450 μg/mL) and linalool (stock concentration 9.85 mg/mL) were also tested for RSY150 and RSY35 [16, 39, 40].

### Growth and viability of *C. albicans* with CNB oil exposure

#### Growth curves

To investigate the effect of CNB oil on growth kinetics of *C. albicans*, an overnight culture of *C. albicans* RSY150 diluted to contain 2.2 × 10^5^ cells/mL (OD_600_ of 0.001) was treated with CNB oil (62.5, 31.25 and 15.1 µg/mL corresponding to 1/2 MIC, 1/4 MIC and 1/8 MIC) in separate wells of a microtiter plate in triplicate and incubated at 30 °C [41, 42]. The OD_600_ was measured at 30 min intervals for 24 h using a BioTek Synergy HTX multi-mode microplate reader (Northern Vermont, USA). Media with CNB oil was used to determine background absorbance for three independent experiments.

#### Cell viability

The cell viability after CNB oil exposure was determined using the methylene blue dye exclusion assay, as reported previously [43]. Briefly, mid logarithmic phase *C. albicans* RSY150 cultures (2.2 × 10^5^ cells/mL) were treated with various concentrations (62.5, 31.25 and 15.1 µg/mL corresponding to 1/2 MIC, 1/4 MIC and 1/8 MIC) of CNB oil at 30 °C for 24 h. Cells fixed with formaldehyde were used as positive controls for staining. The cells were washed and resuspended in phosphate buffered saline (PBS;0.01 M pH 7.4) to ~ 10^7^ CFU/mL, then 100 µL of treated and control cell suspensions mixed with 100 µL methylene blue (0.1 mg/mL stock solution in 2% sodium citrate) and incubated for 5 min at room temperature. Cells were examined using an Eclipse 80i microscope (Nikon) at 40× magnification. A minimum of 100 cells in consecutive visual fields were examined and the percentage of stained cells were calculated using the Nikon SPOT software [44].

To determine colony forming units (CFU), overnight cultures of CNB oil-treated *C. albicans* RSY150 (1/2 MIC, 1/4 MIC, and 1/8 MIC) along with controls were serially diluted (10^3^, 10^4^, 10^5^ cells/mL) and plated in duplicate on YPD agar [45]. Viable colonies were counted and recorded at each specific concentration of CNB oil prepared in triplicate.

### Morphological analysis

Calcofluor white (CFW) at a concentration of 0.01 μg/mL was used as a chitin specific dye [35] to highlight the gross morphology and chitin distribution in CNB oil treated RSY150 along with blood and genital clinical isolates. Cell suspensions at two growth phases each, mid logarithmic (1 × 10^7^ cells/mL) and stationary (2.2 × 10^5^ cells/mL), were treated with MIC and 1/2 MIC of CNB oil and imaged on an Axio Observer Z1 inverted epifluorescence microscope (Oberkochen, Germany) at 63× magnification (λ_ex_ = 365 nm; λ_em_ = 435 nm).

### Hyphal induction

Hyphal induction in *C. albicans* RSY150 was performed according to the literature [46]. Briefly, a yeast suspension (1 × 10^7^ CFU/mL) was prepared from mid logarithmic phase cells in pre-warmed YPD with 10% fetal bovine serum (FBS) and deposited into a 12 well plate with the appropriate amounts of CNB oil to achieve MIC and 1/2 MIC. Control cultures lacked CNB oil. The cells were stained with CFW (0.01 μg/mL) to highlight hyphae and pseudo hyphae after 4 h incubation at 37 °C, and images captured on an AxioObserver Z1 inverted epifluorescence microscope (Oberkochen, Germany). Germ tubes were identified when the cell projection was equal to the size of the blastospore. Results from three independent experiments were reported as average ± standard deviation.

### Ultrastructural and mechanical analysis

The cell surface biophysical properties of CNB oil treated *C. albicans* RSY150 and a clinical isolate from blood were analyzed by Quantitative Imaging (QI™) using a Nanowizard III AFM (JPK Instruments, Berlin, Germany) as described previously [47, 48]. Briefly, cell suspensions (2.2 × 10^5^ cells/mL) from both strains treated either with YPD only (control) or YPD with 1/2 MIC CNB oil for 24 h were deposited onto poly-l-Lysine coated cover slips for 1 h, fixed with formalin and air dried prior to AFM imaging. Cells treated with the fungal wall degrading enzyme, glucanase, served as a positive control. Samples were imaged with silicon nitride cantilevers (HYDRA6R-200NG; Nanosensors, Neuchatel, Switzerland) having calibrated spring constants ranging from 0.03 to 0.062 N/m.

QI force curves (JPK software) obtained at each pixel of a 128 × 128 raster scan were collected using a Z-length of 7 μm and a raster scan of 100 μm/s for QI™. Adhesion and Young’s modulus calculations were made from approximately 16,536 force curves collected from each of five biological replicates. All force curves within a 200 × 200 nm square in the center of the cell were batch processed and histogram data exported from the JPK software using Excel. Adhesion was determined using the distance between the lowest point and baseline of the retract curve and Young’s moduli determined using the Hertz model (JPK software), an estimate of cell envelope elasticity. Surface roughness was measured at the mid-point of the cell using the QI™ height images [48] and cellular volume was calculated using “Ellipsoid” (http://planetcalc.com/) [49] for at least 20 different cells from three different samples.

### Membrane integrity

To assess the integrity of the cell membrane following CNB oil exposure, the cellular content leakage assay [43] was carried out with slight modification. Briefly, a mid logarithmic phase culture of *C. albicans* RSY150 was washed three times and resuspended to ~10^7^ CFU/mL in PBS. Cell suspensions were transferred to a 24 well plate containing CNB oil at MIC, 1/2, 1/4 and 1/8 MIC in PBS, and incubated at 30 °C for 6 h with shaking (200 rpm). CNB oil in PBS served as a blank, untreated cells in PBS as negative controls, and amphotericin B at MIC served as a positive control. Following incubation, cell leakage was analyzed from supernatant diluted 1:10 with PBS based on absorbance at 260 nm (Varian Cary 100 BIO, UV–VIS spectrophotometer; Midland, ON, Canada) against a blank lacking CNB oil. Nucleotides represent one class of leakage components that absorb at 260 nm, for which uracil containing compounds have the highest absorbance. Mean ratios for each treatment from three independent experiments were calculated and compared to that of corresponding untreated samples.

### Cell wall stress

The sensitivity of *C albicans* to cell wall perturbing agents (Congo red (CR) and CFW) after exposure to CNB oil was tested following a previously reported method [6], with minor modifications. Briefly, overnight stationary phase cultures of *C. albicans* RSY150 (2.2 × 10^5^ cell/mL) in YNB complete media, unexposed or exposed to CNB oil at 1/2 MIC and 1/4 MIC, were incubated for 24 h with shaking (200 rpm) at 30 °C in a microtiter plate. Cells were washed with PBS to eliminate oil residues and any carryover effect of the oil to different wells. An aliquot (5 μL) of each was diluted to final densities of 10^5^, 10^4^, 10^3^ cells/mL and were spotted on YNB plates supplemented with one of the following cell wall disrupting agents: CFW (15 μg/mL) or CR (15 μg/mL). The plates were incubated at 30 °C and monitored for *Candida* growth over 3 days. Each experiment was performed in triplicate and colonies counted for statistical analysis and graphical representation.

### Confocal microscopy

The effects of CNB oil treatment on the cell cycle was determined by laser scanning confocal microscopy (LSCM) [35]. RSY150 cells expressing Tub2-GFP and Htb-RFP [35] at mid logarithmic phase (10^7^ cells/mL) were treated with CNB oil, cinnamaldehyde and linalool at MIC and 1/2 MIC for 4 h. Treated and control cells were transferred to glass slides sealed with clean coverslips and imaged at 63 × magnification on a Zeiss LSM 780 system (Carl Zeiss Micro imaging, Oberkochen, Germany) using an argon laser (λ_ex_ = 488 nm; λ_em_ = 512 nm) for Tub2-GFP and HeNe laser (λ_ex_ = 543 nm; λ_em_ = 605 nm) for Htb-RFP. Cells were identified and enumerated in each cycle phase category. Statistical significance was calculated from three independent experiments.

### Statistical analysis

All experiments were performed in triplicate, unless otherwise stated, and GraphPad Prism7 used for statistical analysis. The results were reported as mean ± standard deviation (SD), differences assessed using a two-tailed unpaired *t* test with Welch’s correction at a 95% confidence interval, for which p < 0.05 was considered statistically significant.

## Results

### CNB oil composition

CNB oil analyzed by GC-FID and GC–MS identified several known constituents. The peaks identified by GC-FID were further confirmed with GC–MS and previously reported RI [32, 34]. The composition of our CNB is shown in Additional file 1: Table S1 (see Additional file 2: Figure S1), for which compounds are listed in the order of elution on the HP-5 column. The major compounds (concentrations > 2.0 as relative % peak area from GC-FID and GC–MS) were E-cinnamaldehyde (74%), α-caryophyllene (5.3%), linalool (3.9%) and E-cinnamyl acetate (3.8%). Minor components with known function included limonene (2%), eugenyl acetate (0.6%), α-pinene (0.3%) and benzyl benzoate (0.6%). Another minor constituent was p-cymene (1.4%).

### RSY150 and clinical isolates of *C. albicans* respond differently to CNB oil

The relative susceptibilities of the RSY150 strain and clinical isolates to CNB oil were determined using a broth microdilution method. The planktonic growth of *C. albicans* lab strains (ATCC 64548 and RSY150) was effectively inhibited by 125 μg/mL CNB oil, whereas the clinical strain isolated from blood and the ATCC 10231 strain were more resistant and required higher concentrations (250 μg/mL) of CNB oil to produce an inhibitory effect. This clinical strain isolated from blood was selected for detailed analysis and further experiments. Clinical isolates from blood and genital infections displayed varying susceptibility to CNB oil. In general, a blood isolate was resistant to CNB oil with a MIC of 500 μg/mL, whereas one genital strain was more resistant to CNB oil with a MIC at 1000 μg/mL and the other more susceptible with a MIC of 125 μg/mL. The data is summarized in Table 2. RSY35, a mutant strain for microtubule motor protein Kar3, showed growth inhibition at 125 μg/mL of CNB oil. Similarly, the growth of RSY150 and RSY35 was inhibited by cinnamaldehyde with a MIC of 112.5 μg/mL. Both strains showed identical response to linalool with MICs at 2.46 mg/mL.

**Table 2:** Number of reference and clinical strains at various MIC values

CNB oil (µg/ml)	Blood	Genital	RSY150	RSY35	ATCC 10231	ATCC 64548
1000		1				
500	1					
250	*1					
125	3	**1	1	1	1	1

*Used as representative clinical strain in all further experiments

**Tested for chitin staining

### Dose dependence of *C. albicans* RSY150 growth, viability and membrane integrity

**Fig. 1:**
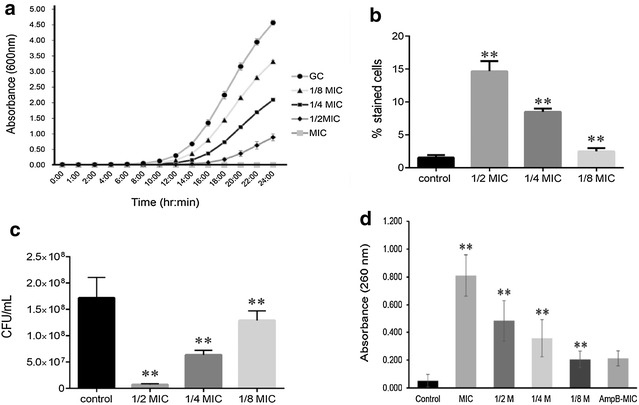
CNB oil decreased cell viability and compromised cell membrane integrity in RSY150. **a** Growth curves of RSY150 exposed to 125, 62.5, 31.25 and 15.125 µg/mL of CNB oil, with absorbance measured at 30 min intervals to show the antifungal time course of the CNB oil. **b** Methylene blue staining of cells exposed to CNB concentrations as in (**a**) showed decreased cell viability. **c** Corresponding CFU counts of viable CNB exposed cells as compared to control. **d** Leakage of cellular content as a function of absorbance at 260 nm following exposure to CNB oil at concentrations as in (**a**) confirm a dose dependent compromise of the cell membrane in (**a**). GC indicates growth controls of cells incubated without CNB oil. Double asterisks represent statistical significance (p < 0.05)

To determine the dose dependence of CNB oil over a 24 h period, growth curves were constructed at different concentrations of CNB oil (15.625–125 µg/mL) in YPD media. Growth inhibition was dose dependent with complete inhibition of growth at MIC (125 µg/mL) and increased growth at progressively lower sublethal concentrations: 62.5 μg/mL (1/2 MIC), 31.25 μg/mL (1/4 MIC) and 15.625  µg/mL (1/8 MIC) (Fig. 1a), used for subsequent experiments.

The viability of cells cultured to logarithmic phase after treatment for 4 h with different concentrations of CNB oil (125–62.5 µg/mL) were assessed using the vital dye, methylene blue (Fig. 1b), which enters both live and dead cells. Since live cells are able to reduce the dye [50, 51] they become colorless, whereas dead cells remain blue. The cells were non-viable following CNB oil treatment, confirmed by CFU for each CNB oil treatment (1/2, 1/4 and 1/8 MIC and control). There was a significant reduction in the number of colonies treated with 1/2 MIC CNB oil in comparison to cells treated at 1/4, 1/8 MIC and control (Fig. 1c).

Membrane disruption in CNB oil treated RSY150 cells was assessed as leakage of cellular contents (OD_600_ = 260 nm) in the supernatant [43]. The amount of leaked cellular content increased as a function of CNB oil concentration, from 1/8 MIC to MIC (Fig. 1d), showing that compromised cell membrane integrity is dose dependent. The positive control amphotericin B showed leakage at MIC.

### Sublethal doses of CNB oil altered chitin distribution in *C. albicans*

**Fig. 2:**
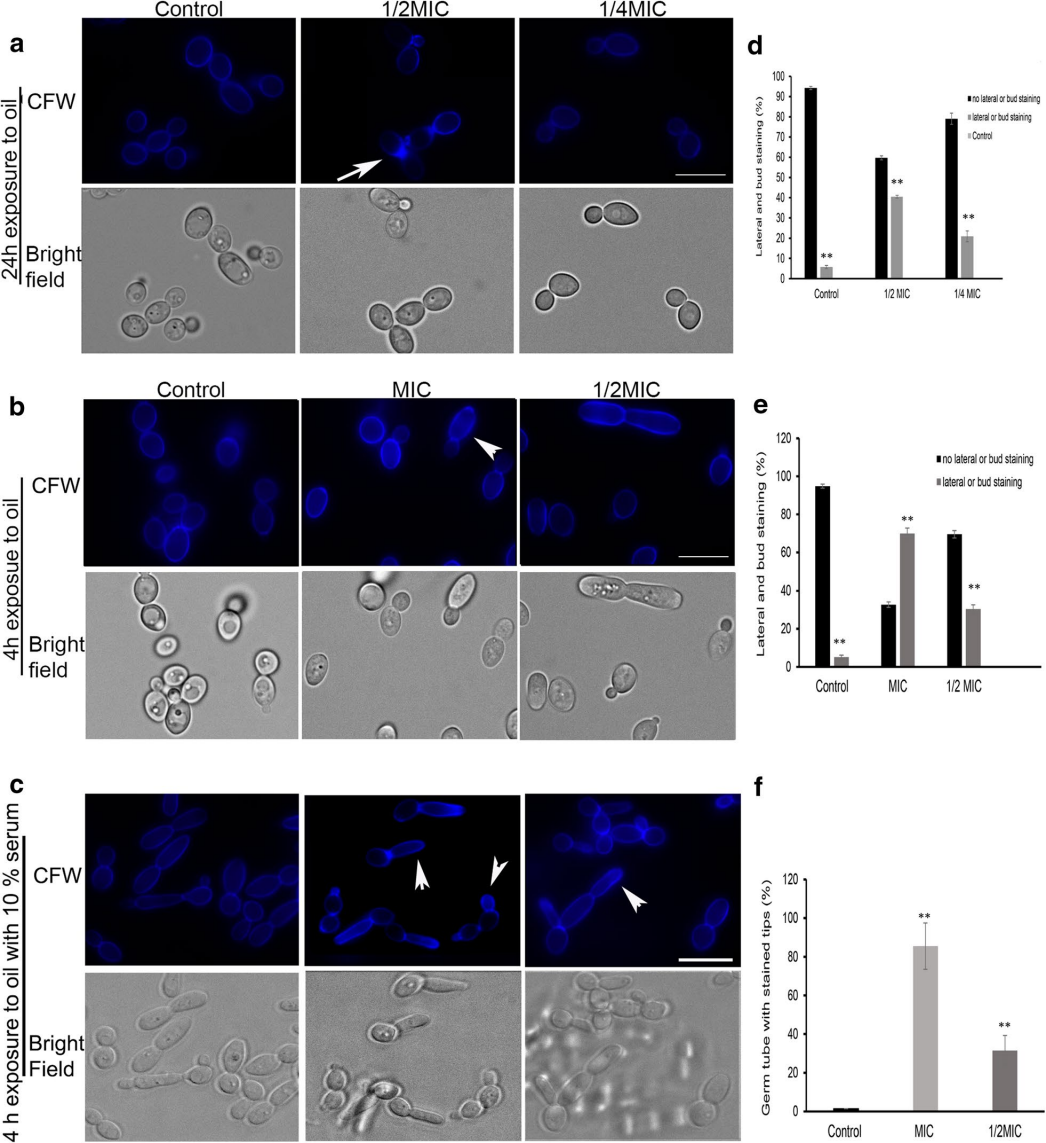
CNB oil exposure induces a cell wall stress response with altered chitin distribution in RSY150. Epifluorescence (top panel) and bright field (bottom panel) images of CFW stained cells. Cells were grown in YPD in (**a**) and (**b**) while cells in (**c**) were grown in YPD plus 10% serum for hyphal induction. **a** RSY150 cells were incubated for 24 h with and without 1/2 and 1/4 MIC CNB oil in YPD media and stained with CFW. **b** Mid log phase RSY150 were exposed to MIC and 1/2 MIC for 4 h stained with CFW and imaged. **c** Cells grown in YPD with 10% serum were exposed to the same concentration as in (**b**) for 4 h, stained with CFW and imaged. (**d**, **e**, **f**) are bar graphs showing enumeration of cells in (**a**, **b** and **c**) respectively, where double asterisks indicate p < 0.05. Bars = 5 μm

To determine morphological changes, we analyzed bright field images of *C. albicans* in stationary and logarithmic phases exposed to sublethal concentrations of CNB oil for 24 h. Stationary phase *C. albicans* RSY150 cells exposed to sublethal CNB were oval and swollen, with distinct changes in chitin distribution, but with normal budding compared to control cells (Fig. 2a, d). Epifluorescence images of stationary phase control RSY150 show an even distribution of chitin around the cell wall and septal region (Fig. 2a, d). In contrast, cells treated with CNB oil at 62.5 μg/mL (1/2 MIC) had an increase in chitin content in the lateral cell wall and more intense staining at septal regions (Fig. 2a, d). This effect was absent at lower concentrations (31.625 μg/mL; 1/4 MIC) of CNB oil.

Similarly, the effects of CNB oil on metabolically active cells were distinct. Logarithmic phase cells exposed to CNB oil for 4 h at MIC and 1/2 MIC had intense chitin staining in the bud neck region and uneven lateral cell wall staining (Fig. 2b, e) compared to control cells that stained evenly at the bud and lateral wall. We also observed an increased frequency of elongated cells during exposure to CNB oil at MIC, which became less prominent at 1/2 MIC (Additional file 3: Figure S2a, b). The clinical isolate from blood, on the other hand, showed overall stronger staining than RSY150, with increased intensity at the bud neck upon exposure to CNB oil at MIC and 1/2 MIC (Additional file 4: Figure S3a). In contrast, the genital clinical isolate having a similar MIC to RSY150 showed a normal chitin distribution (Additional file 4: Figure S3b).

Based on the altered phenotype during CNB exposure at sublethal levels, we studied the impact of CNB on hyphal growth. During hyphal induction (10% serum) in the presence of CNB oil, growing hyphae had intense chitin staining at their growing tips. The intensity of chitin staining was higher in cells treated at MIC and decreased progressively as a function of CNB oil concentration, at 1/2 MIC (Fig. 2c, f). At 1/4 MIC CNB the intensity of CFW staining was comparable to control, however hyphal width remained the same.

### Sublethal CNB oil induces cell wall remodelling in *C. albicans*

**Fig. 3:**
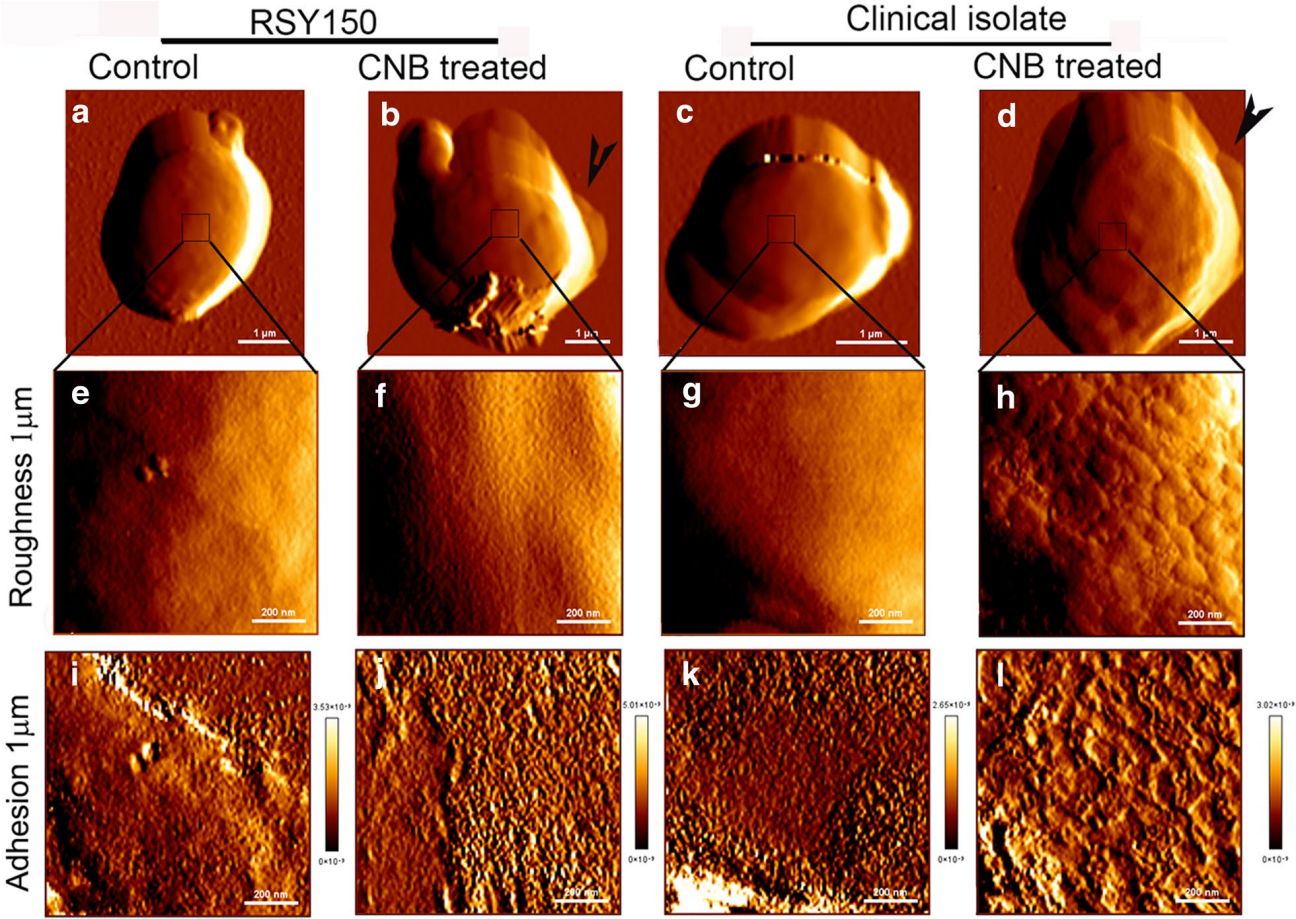
Surface remodelling of RSY150 *C. albicans* exposed to 1/2 MIC CNB oil. RSY150 and the clinical isolate from blood were treated with 1/2 MIC (62.5 μg/mL) CNB oil for 24 h, deposited and fixed on PLL-coated cover slips for AFM imaging. Images (1 μm; 128 px^2^) of control and CNB oil treated cells are at low resolution for control (**a**, **c**) and treated (**b**, **d**) *C. albicans* showing leakage (arrow heads) in treated cells. High resolution (1 μm) height images of the cell surface (boxed area) for control (**e**, **g**) and CNB treated (**f**, **h**) *C. albicans* reveal increased surface roughness in the treated clinical isolate as compared to the treated RSY150 strain. (**i**, **j**, **k**, **l**) High resolution (1 μm) adhesion maps from corresponding height images. Bar a–d = 1 μm; Bar e–l = 200 nm

AFM is a powerful tool for imaging ultrastructural changes at the cell surface and probing the associated biophysical properties. We chose QI™ mode, which records a large number of force curves (n = 16,536; 128 pixel^2^ resolution) for a detailed analysis. Representative QI™ images of RSY150 and a clinical isolate of *C. albicans* exposed to sub lethal concentrations of CNB oil, along with comparable controls, are shown in Fig. 3a–d. Changes in RSY150 and the clinical isolate after CNB treatment are summarized in Table 3. There was a clear difference between the CNB treated RSY150 and the clinical isolate, with 50% of the RSY150 cells showing leakage and exfoliation while the clinical isolate showed leakage with increased surface roughness. The exfoliation in these cells was usually limited to a small area on the cell in the form of an aberration, while the rest of the surface showed little change. The clinical isolate exposed to CNB oil had rough cell surfaces, with or without leakage.

**Table 3:** Various changes to *C. albicans* cells with CNB oil exposure revealed by AFM

*C. albicans* treated with CNB oil (total # of cells)	Leakage only	Exfoliation only	Leakage and exfoliation	Surface roughness only	Leakage and surface roughness
RSY150 (n = 24)	9	3	12	–	–
Clinical strains (n = 21)	1	1	1	5	13

Control cells had no visual changes

*Candida albicans* RSY150 and the clinical isolate displayed cell swelling and substantial cell wall structural and mechanical changes in response to CNB oil (Fig. 3). QI™ AFM of RSY150 (n = 24) and a clinical isolate from blood (n = 21) exposed to 1/2 MIC of CNB oil showed obvious changes in cell volume, roughness, surface adhesion and cell wall elasticity.

Images of untreated RSY150 (Fig. 3a, c) revealed a normal oval cell with a height of 1.65 ± 0.19 μm and cell volume of 9.41 ± 1.7 μm^3^, which significantly (p < 0.0001) increased to a height of 2.18 ± 0.31 μm and volume of 18.4 ± 4.7 μm^3^, respectively, following CNB oil treatment. The change in height (2.07 ± 0.29 μm vs. control 1.83 ± 0.27 μm) (p = 0.01) and volume (volume: treated 12.7 ± 5.9 μm^3^ vs. control 16.2 ± 4.7 μm^3^) (p = 0.047) of the clinical isolate was significant with CNB oil treatment, but less pronounced than that of RSY150 with similar treatment.

Control cells of both strains (Fig. 3e, g) revealed a smooth and homogeneous surface. There was a significant increase (p = 0.015) in cell surface roughness (5.4 ± 1.0 nm) of the clinical isolate following CNB oil exposure compared to control (2.7 ± 1.2 nm), whereas the RSY150 (Fig. 3e–h) showed no significant change (p = 0.2432) compared to control cells (treated 4.9 ± 1.08 nm vs. control 3.52 ± 1.77 nm). These results are summarized in Table 4.

**Table 4:** Comparison of ultrastructural properties of RSY 150 and the clinical isolate from blood

	RSY150		Clinical isolate	
	Control	Treated	Control	Treated
Height (nN)	5.5 ± 0.3	7.1 ± 0.5	5.7 ± 0.4	7.6 ± 0.6
Volume (μm^3^)	9.4 ± 1.7	18.4 ± 4.7	16.2 ± 4.7	12.7 ± 5.9
Roughness (nm)	3.5 ± 1.8	4.9 ± 1.1	2.7 ± 1.2	5.4 ± 1.0

For accurate adhesive measurements of the cell surface, 1 μm QI™ AFM images (128 px^2^ resolution) were captured at the center top of the cells to eliminate artifacts from cell curvature. The adhesive properties of both RSY150 (treated 7.1 ± 0.5 nN vs. control 5.5 ± 0.3 nN) and the clinical isolate (treated 7.6 ± 0.6 nN vs. control 5.7 ± 0.4 nN) were significantly increased (RSY150 p = 0.0016, clinical isolate p = 0.0025) in response to CNB oil. Figure 3b, f, j and d, h, l shows whole cells, high resolution images, roughness and adhesive forces on the center top of RSY 150 and clinical isolates, respectively, grown for 24 h in the presence of sublethal (62.5 μg/mL) CNB oil. Exposure to CNB oil altered cell shape, increased the average cell volume and roughness (Fig. 3j, f), with a twofold increase in elasticity for RSY150 (treated 7.8 ± 0.9 GPa vs. control 4.7 ± 0.4 GPa) and the clinical isolate (treated 15.0 ± 2.7 GPa vs. control 8.1 ± 0.7 GPa) compared to their respective controls. Table 5 shows a summary of these differences in RSY150 and the clinical isolate.

**Table 5:** Comparison of biophysical properties of RSY150 and the clinical isolate from blood

	RSY150		Clinical isolate	
	Control	Treated	Control	Treated
Adhesion (nN)	5.5 ± 0.3	7.1 ± 0.5	5.7 ± 0.4	7.6 ± 0.6
Elasticity (GPa)	4.7 ± 0.4	7.8 ± 0.9	15.0 ± 2.7	8.1 ± 0.7

### CNB oil exposure increases *C. albicans* sensitivity to cell wall disrupting agents

**Fig. 4:**
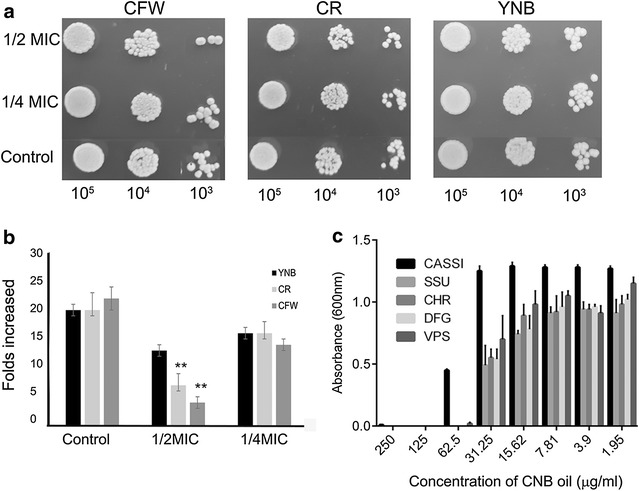
CNB treated RSY150 *C. albicans* exhibit a cell wall stress response. Logarithmic phase RSY150 cells were treated with 1/2 and 1/4 MIC CNB oil or without oil for 24 h. **a** Treated and control cells at 10^5^, 10^4^ and 10^3^ CFU were spotted on YNB complete media agar containing CFW and CR and examined after 2 days. Treated cells showed greater sensitivity to cell wall stressors. **b** The number of colonies on the CR and CFW plates show a dose dependent effect that decreases as a function of oil concentration. The 1/2 MIC CNB oil was more effective than 1/4 MIC, with a significant difference ** between 1/2 MIC oil treatment compared to untreated controls (p < 0.05). The cell count was normalized with treated cells grown on YNB media agar only. **c** Wild type background strain (CaSS1) and cell wall defective mutants (*SSU81, CRH, DFG* and *VPS*) were exposed to a twofold increasing dilution of CNB oil in a MIC assay, showing overall sensitivity to the CNB oil and indicating a moderate cell wall stress response

In order to determine whether cell wall integrity was compromised by CNB, RSY150 were exposed to cell wall perturbing agents CR and CFW at 1/2 MIC and 1/4 MIC. Higher sensitivity, reflecting compromised cell wall integrity, was observed in CNB treated cells in comparison to control (Fig. 4a) at 1/2 MIC, progressively decreasing to 1/4 MIC (Fig. 4b). As positive controls, we analyzed mutants lacking genes related to cell wall integrity and to wall organization and biogenesis for their sensitivity to CNB oil treatment. The mutants tested had the following knockouts: *VPS28*, a VPS factor required for vacuolar protein sorting; *CRH11*, encoding a GPI-anchored cell wall transglycosylase; *SSU81*, encoding a function involved in oxidative stress, cell wall biogenesis, and morphogenesis; and *DFG5*, encoding an N-linked mannoprotein of the cell wall and membrane. All these mutants exhibit generalized sensitivity to CNB oil, with lower MIC than the wild type strain (CaSS1), further verifying an impact of CNB on cell wall integrity (Fig. 4c). The associated MIC decreased two-fold, from 125 μg/mL to 61.5 μg/mL, for all mutants.

### CNB oil induces spindle defects in *C. albicans* at MIC and sub-MIC

**Fig. 5:**
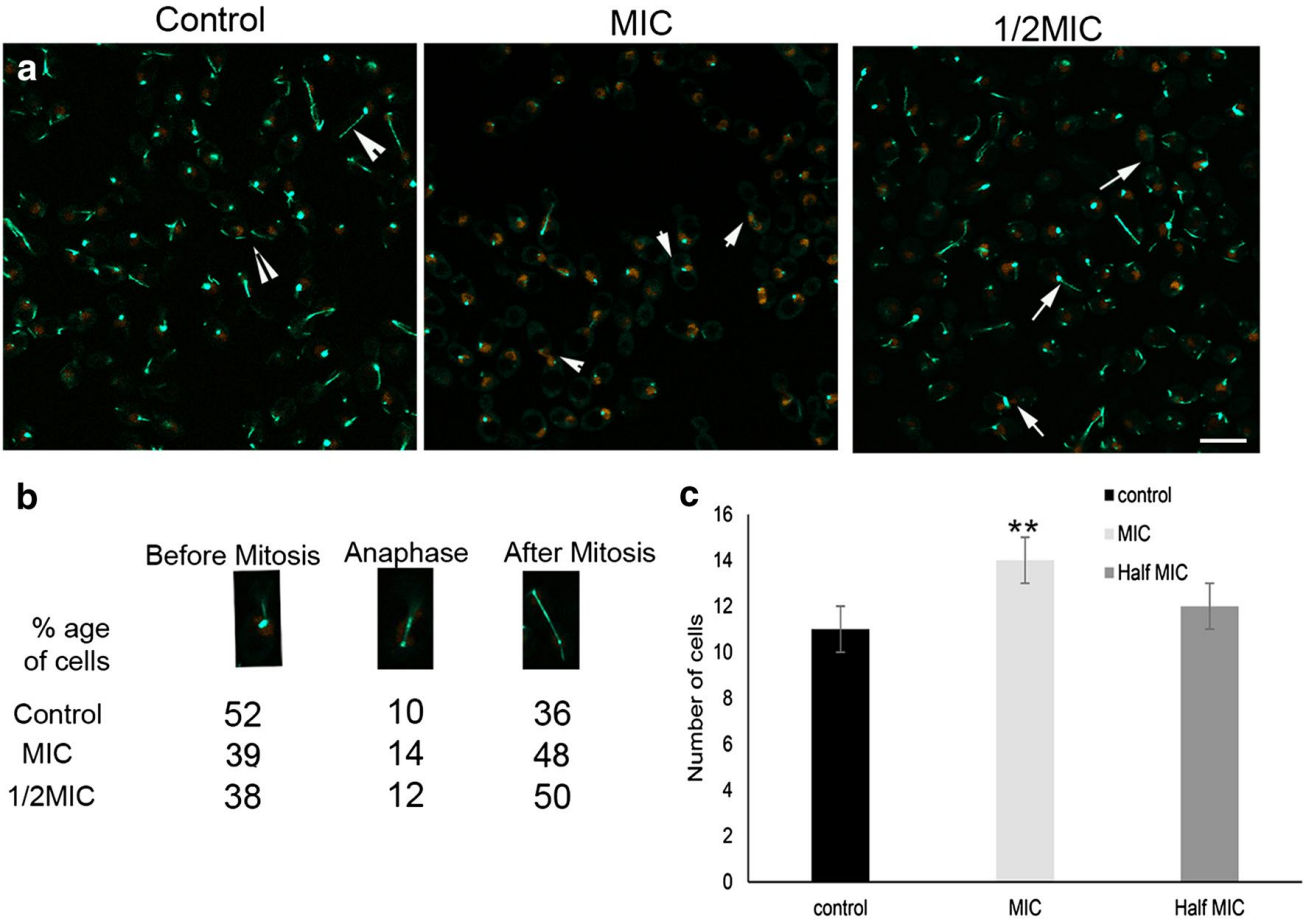
CNB oil exposure in RSY150 *C. albicans* caused spindle defects in RSY150 and a delayed cell cycle. Mid logarithmic phase RSY150 cells were treated with MIC and 1/2 MIC oil for 4 h and imaged live by LSCM (Tub2-GFP λ_ex_ = 488 nm; λ_em_ = 512 nm and Htb-RFP λ_ex_ = 543 nm; λ_em_ = 605 nm). RSY150 were examined for nuclear and spindle localization in budding cells. **a** Spindle morphologies of MIC and 1/2 MIC CNB oil treated cells and untreated control. Control cells showed normal spindle formation during mitosis (arrow heads) whereas the MIC treated cells had a majority of dividing cells lacking a mitotic spindle (small arrow heads). Cells treated with 1/2 MIC of CNB oil had diminished spindle defects. Despite the spindle defect, the cells completed mitosis. Images are representative from three independent experiments. Bar = 5 μm. **b** Cell cycle analysis of cells treated with MIC and 1/2 MIC CNB oil and untreated controls. Of cells treated at MIC CNB oil, 14% were in anaphase in comparison to 10% for control cells, whereas 12% of cells exposed to 1/2 MIC CNB oil were in anaphase. **c** The number of cells counted for treated and control cells in anaphase were plotted. Double asterisks represent statistical significance (p < 0.05). For each treatment 250 cells were analyzed

The RSY150 strain expressing both Tub2-GFP-and Htb-RFP was imaged by LSCM to visualize the mitotic spindle in dividing cells exposed to CNB oil. In untreated cells, the spindle varied in length for the control RSY150 strain, but it consistently spanned the distance between mother and daughter cells (Fig. 5a) and nuclear migration was normal. In contrast, these well-defined mitotic spindles were missing in RSY150 cells exposed to CNB oil at MIC. Furthermore, Tub2-GFP fluorescence in these cells consistently failed to show the wild type pattern of spindle formation between mother and daughter cells (Fig. 5b). Instead, Tub2-GFP often had reduced fluorescence in cells undergoing nuclear division, with only several fluorescence spots visible, indicating disruption of the mitotic spindle. Cells exposed to sub MIC CNB oil had reduced spindle defects, with most of the cells in recovery phase and observed as fluorescent patches or aggregates of tubulin (Fig. 5c).

RSY150 cells grown to the mid logarithmic phase and exposed to oil for 4 h were imaged by LSCM to view patterns of nuclear division and tubulin distribution in the parent cells and the bud. Budding cells with a single nucleus were considered to be pre-mitosis, representing those in the G1 or S phase. Cells with nuclei traversing the parent and budding cells were considered to be in the anaphase stage of mitosis. Finally, cells having separate nuclei in both parent and budding cells were considered to be at a post-mitotic phase, G2/M. For RSY150 control cells, 52% of the population was in the G1 or S phase; 36% of the cells had undergone mitosis; and the final 10% were in anaphase (Fig. 5b, c). On the other hand, RSY150 treated with MIC of CNB oil displayed noticeable differences in cell cycle distribution, with 39% having not begun mitosis, 48% having completed mitosis, and 14% in anaphase, still undergoing nuclear division (Fig. 5c). Cells treated with 1/2 MIC were at GI phase (38%), whereas the dominant number of cells were at G2/M phase, with no change in the number of cells undergoing anaphase. Cells treated at MIC with CNB oil showed extended cells with pseudohyphal formation, however the effect was less pronounced in cells treated at 1/2 MIC, where large budding cells were observed (Additional file 3: Figure S2a, b). The data clearly indicate that CNB oil induces tubulin depolymerisation leading to cell cycle arrest at anaphase.

Cinnamaldehyde treated *C. albicans* showed similar morphological features to CNB oil treated cells (Additional file 5: Figure S4), with depolymerized tubulin, resulting in an absence of mitotic spindles. Overall the effect was more pronounced in cinnamaldehyde treated cells as compared to those treated with CNB oil. Cells exposed to cinnamaldehyde at MIC showed a significantly increased percentage of cells in anaphase (15.8%) as compared to control (9.6%), whereas RSY150 cells exposed to linalool were arrested at the G1 phase of the cell cycle, as previously reported [20]. There was complete disruption of the cell wall at MIC after 4 h, with cells having depolymerized tubulin compared to controls, however the cells showed some tubulin fluorescence at 1/2 MIC, with intact cell walls (Additional file 5: Figure S4).

## Discussion

The cell wall of *C. albicans* is the first line of defense against the host immune system and toxic agents, modulating fungal interactions and maintaining cell integrity [52–55]. The *C. albicans* yeast cell wall contains three main components: mannoproteins (~ 39%), β-glucans (~ 59%), and chitin (~ 2%), that contribute to a highly dynamic molecular architecture which is continuously remodelled in response to cell surface interactions [53–55]. The cell wall integrity (CWI) pathway compensates for damage caused by environmental stress, signaling through the MAPK cascade to mediate cell wall biosynthesis and actin organization, regulating cell cycle progression and other necessary events [56–59]. In response to cell wall stressors, for example the antifungal drug caspofungin which is a non-competitive inhibitor of β-1,3-glucan synthase [60–62], activation of the CWI pathway can reinforce the cell wall by increasing chitin levels in *Candida* [63] and *C. glabrata* [64].

### Cell wall and membrane stress

CNB oil treated RSY150 cells in the stationary phase had an irregular chitin distribution found on the lateral wall of budding cells (Fig. 2a). Such effects became pronounced in metabolically active cells grown in the presence of 10% serum (Fig. 2c, f), with intense chitin staining at the hyphal tip. Similar staining patterns have been reported for cells exposed to high sugar or salt concentrations [23], for which hypo-osmotic pressure induced chitin synthesis or polymerization at the growing hyphal tip. Consistent with this idea is the larger cell volume of RSY150 (Fig. 3) and sensitivity of the *SSU18* mutant (Fig. 4c) exposed to CNB oil. Cell wall reinforcement with chitin in response to stress is a well-known phenomenon in yeast [62, 64–67]. It is known that most resistant clinical strains have a higher overall chitin content, reinforcing the cell wall and making them more resistant to cell wall targeted antifungals [68]. As expected, the metabolically active clinical isolate showed more chitin than RSY150 (Additional files 3, 4: Figures S2, S3) for both control and treated cells, confirming cell wall reinforcement in the clinical strain.

Chitin plays an important role in defining the cell wall nanomechanical properties [25, 27, 69], and β-1,3 D-glucan is critical in maintaining cell shape, mechanical rigidity and resistance to osmotic pressure [4, 70]. Exposure to CNB oil produced osmotically fragile, swollen cells with an uneven chitin distribution and increased elasticity, consistent with prior studies [25, 27, 52, 59]. The elasticity values reported in this study are high in comparison, reflecting cross linking from fixation [71]. Inhibition of β-1,3-d-glucan synthase by cinnamaldehyde in *S. cerevisiae* or by caspofungin in *C. albicans* results in reduced cell wall β-1,3-glucan [22] and increased cell wall chitin content, respectively [25]. Furthermore, mutants defective in chitin and β-glucan cross linking (*chr1chr2Δ*) have cell wall elasticity that varies with architecture and composition [60].

In addition to cell wall chitin and glucans, the surface of *C. albicans* is decorated with proteins called adhesins that play a pivotal role in cell communication, adhesion and microbial infection [72, 73]. Many adhesins are mannoproteins that are homogenously dispersed on the cell surface [74, 75]. Cell wall stress, temperature variation, exposure to antifungal agents, host interaction and biofilm formation have been shown to alter the adhesive properties of cells [67, 76]. Adhesive forces in this study are based on the interaction between the cell surface and the hydrophilic, negatively charged silicon nitride AFM tip. Exposure to CNB oil gave rise to a moderate increase in cell adhesion, which may be attributed to cell wall remodelling, as evidenced by concomitant changes in roughness. Changes in adhesion may reflect loss or rearrangement of adhesins, or reorganization of surface sugars [25, 27, 29].

Cell wall stress was characterised not only by changes in chitin distribution and expression, but also polarized growth and moderate sensitivity to cell wall perturbing agents, leading to cell cycle arrest and fungal death [57]. Congo Red and Calcofluor White target chitin and β-1,3-glucan synthases to produce a cytokinetic defect leading to cell cycle arrest [77, 78]. A delayed or arrested cell cycle results in polarized growth with formation of pseudohyphal cells [79–81], as characterized by depolymerized microtubules [35, 82, 83]. Such a morphology was observed in 30% of the cell population exposed to CNB oil, along with defects in microtubule polymerization.

### Spindle defects

Microtubules are central to cell division, forming the mitotic spindle and coordinating nuclear movement [82, 84, 85]. In *Candida*, the pre-mitotic movement of the nuclei through the bud neck and subsequent separation of nuclei are coordinated by microtubules [86]. In normal cells, nuclear division is complete when the buds are still small, such that larger budding cells are never observed without the nucleus [86]. In the control cells, two distinct subcellular microtubule structures were observed, one traversing the mother and budding cells and the other only in the mother cells. Anaphase and G2 cell cycle arrest, as well as a more pronounced filamentous growth, were apparent following CNB exposure (Fig. 5a, c). Cell cycle defects associated with microtubule perturbation were readily visualized in CNB treated RSY150 in which Htb-RFP and Tub2-GFP highlighted the nucleus and microtubules, respectively. Four hours following treatment with CNB at MIC, log phase cells had ablated mitotic spindles, observed as fluorescent patches or aggregates of tubulin (Fig. 5a). Large-budding cells had nuclear staining restricted to the mother cell (Fig. 5a, arrow). These results are consistent with previous observations in which tubulin polymerization inhibitors and Δ*kar3/Δkar3* mutants had similar phenotypes [35, 85]. We observe anaphase arrest in cells exposed to CNB at MIC, with 14% of the cells in anaphase as compared to 10% in the control, an effect which is reduced at 1/2 MIC. Cells treated at MIC cinnamaldehyde had 15% of cells in anaphase, slightly less than the Δ*kar3/Δkar3* mutant that had 17% of cells in anaphase and exhibited polarized growth [35]. Kar3 may mediate microtubule sliding during nuclear fusion and possibly mitosis, interacting with spindle microtubules to produce an inwardly directed force on the poles that antagonizes the CIP8 and KIP1 outward force [36]. The Δ*kar3/Δkar3* mutant (RSY35) is no more resistant to CNB oil than wild type, suggesting that Kar3 is not likely the target of CNB oil.

We observed pseudohyphal growth in CNB oil treated RSY150 at MIC, an effect which became less prominent at 1/2 MIC, a common response of yeast experiencing cell wall stress [87]. For instance, toxins and chemicals that arrest cells in the S phase or mitosis display polarized growth due to activation of the CWI pathway [57, 88, 89]. The CWI pathway activates the MAPK cascade to manage stress, and its components are key elements in controlling the cell cycle. There are three MAPK pathways, namely; the pheromone response pathway, the high osmolality glycerol (HOG) pathway and the *PKC1*-mediated pathway [88, 89]. The increased sensitivity of the *SSU81* knock-out mutant (Fig. 4c) suggests that the HOG pathway is activated in the presence of CNB oil. Under stress there is cross talk between these pathways and cell cycle checkpoints, resulting in a delayed cell cycle [88] and a mixture of yeast and pseudohyphal cells (Additional file 3: Figure S2a, b).

### Impact of CNB oil components

The multisite impact of CNB oil can be attributed to both its major (cinnamaldehyde) and minor components. Cinnamaldehyde has been shown to compromise cell membrane and wall integrity [16], which we observe in this study along with a pronounced spindle defect and anaphase arrest. Minor components, which include limonene (2%), eugenyl acetate (0.6%), linalool (3.9%) and benzyl benzoate (0.6%), also contribute to CNB oil antifungal activity. The synergistic, additive or antagonist activity of these components to modify the overall effect of the oil has yet to be explored. However based on previous reports, these components target the cell membrane and modulate its function, including fluidity and permeability, leading to cell death [16–20]. Limonene (2%) has been reported to affect the cell wall in *S. cerevisiae* [90, 91] and p-cymene (1.4%) inhibits germ tube formation and alters cell membrane integrity [92]. Eugenyl acetate and linalool exhibit anti-virulence activity by inhibiting germ tube formation, and linalool has been reported to arrest cell cycle in the GI phase [20]. Such studies used higher (mg) concentrations of CNB oil components to produce their effects in comparison with their presence in CNB oil at only 0.6-5%. There remains the possibility of synergies between the CNB oil components.

### CNB oil as an antifungal

We propose that the main component of CNB oil, namely cinnamaldehyde (75%), is responsible for the majority of the cell envelope defects observed in this study. The cell envelope mutants and cell stressor assays demonstrate that the cell wall is moderately affected, reinforced by chitin. Further, the sensitivity of all the cell envelope mutants indicates multiple targets for CNB oil, consistent with its chemical heterogeneity. Our data reports for the first time the depolymerisation of β tubulin leading to cell cycle arrest at MIC of CNB oil. The mitotic spindle defect, a novel finding of this study, we attribute to cinnamaldehyde. The effect was strong at MIC and reduced at 1/2 MIC in cells exposed to either CNB oil or cinnamaldehyde. We propose that membrane disruption may lead to aberrant actin filaments, by virtue of actin’s association with the membrane, and microtubule depolymerisation that ultimately disrupts the mitotic spindle, causing cell cycle arrest. This notion is supported by a finding in *S. cerevisiae*, for which both cytoplasmic microtubules and actin filaments are needed for spindle orientation [93], but further study is required to determine the role of actin in tubulin depolymerisation.

In summary, we suggest that CNB exerts its antifungal effect by targeting multiple cellular sites, including the cell wall, membrane and cell cycle machinery, ultimately leading to cell death induced by cell cycle arrest, a mechanism which requires further study.

## Conclusions

In conclusion, the CNB oil used in this study consists of a mixture of terpenoids, phenols and aldehydes in varying amounts which target multiple cellular sites. Taken together, our data suggest essential oils may serve as antifungal alternatives or could be used in combination with synthetic antifungal agents to combat antifungal resistance.

## Additional files

**Additional file 1: Table S1.** Chemical composition of CNB oil.

**Additional file 2: Figure S1.** Gas chromatogram of CNB oil. High deviation in RI values for E-cinnamaldehyde and α-caryophyllene result from its high concentration in the CNB oil, resulting in a non-Gaussian behaviour of the peak, and low concentration with late elution, respectively.

**Additional file 3: Figure S2.** (a) Cell cycle stress induced pseudohyphae in CNB oil exposed RSY150. Mid log phase RSY150 after 4 h exposure to CNB oil at MIC and 1/2 MIC, were stained with CFW. Images are epifluorescence (top panel) and bright field (BF; bottom panel). Bar = 5 μm. (b) Quantification of cells in control, MIC and 1/2 MIC from (a). Double asterisks represent p < 0.05.

**Additional file 4: Figure S3.**(a) Clinical isolate exposed to CNB oil showed increased chitin content. The clinical isolate from blood at log phase after 4 h exposure to CNB oil at MIC and 1/2 MIC were stained with CFW. Images represent CFW (top panel) and bright field (BF; bottom panel). Bar = 5 μm. (b) Genital clinical isolate with comparable MIC to RSY150 showed a normal chitin distribution.

**Additional file 5: Figure S4.** Spindle morphology of cinnamaldehyde and linalool treated *C. albicans*. Live LSCM of mid log phase cells after 4 h exposure to MIC and 1/2 MIC of cinnamaldehyde and linolool. Cinnamaldehyde treated *C. albicans* at MIC showed a similar spindle morphology of those treated with CNB oil at MIC, whereas linalool treated cells showed a complete absence of tubulin at MIC, with decreased cell size. At 1/2 MIC for both cinnamaldehyde and linalool, tubulin expression appeared as fluorescent spots near the nucleus. Bar = 5 μm.
