# Supplementary figures and images for: Cinnamomum zeylanicum bark essential oil induces cell wall remodelling and spindle defects in Candida albicans
Source: Fungal Biol Biotechnol. 2018 Feb 9;5:3. doi: 10.1186/s40694-018-0046-5 (PMC5807769; doi:10.1186/s40694-018-0046-5)

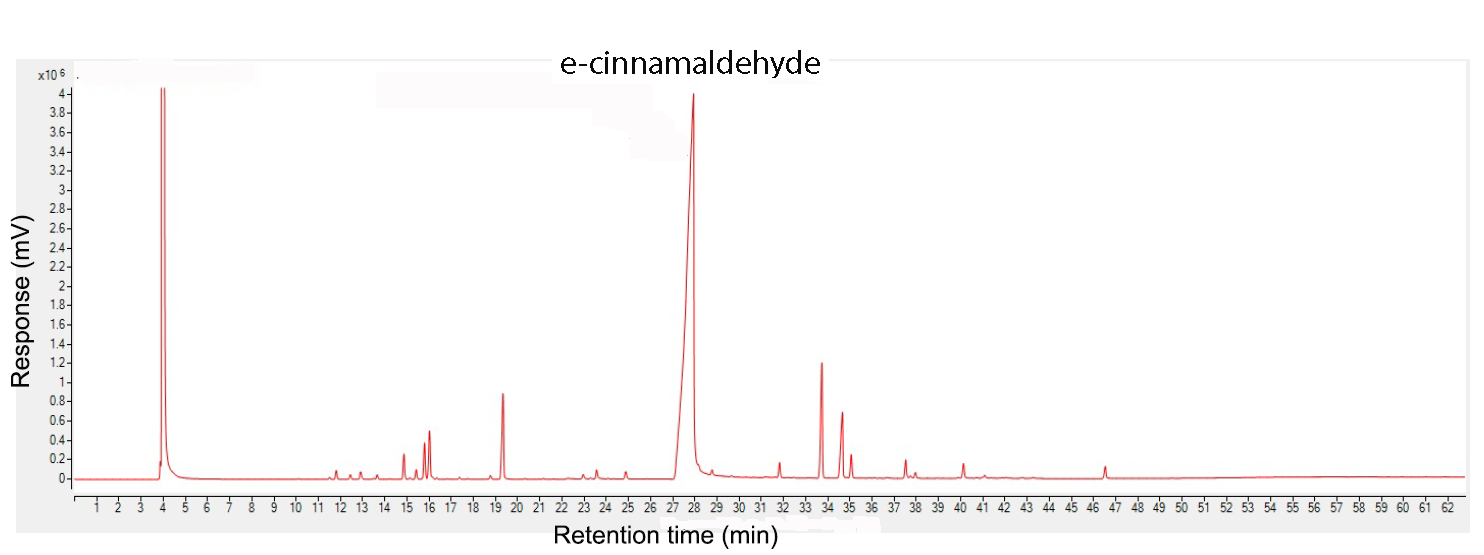

Supplement: Supplementary file 2 — Additional file 2: Figure S1. Gas chromatogram of CNB oil. High deviation in RI values for E-cinnamaldehyde and α-caryophyllene result from its high concentration in the CNB oil, resulting in a non-Gaussian behaviour of the peak, and low concentration with late elution, respectively. [file 40694_2018_46_MOESM2_ESM.tif]

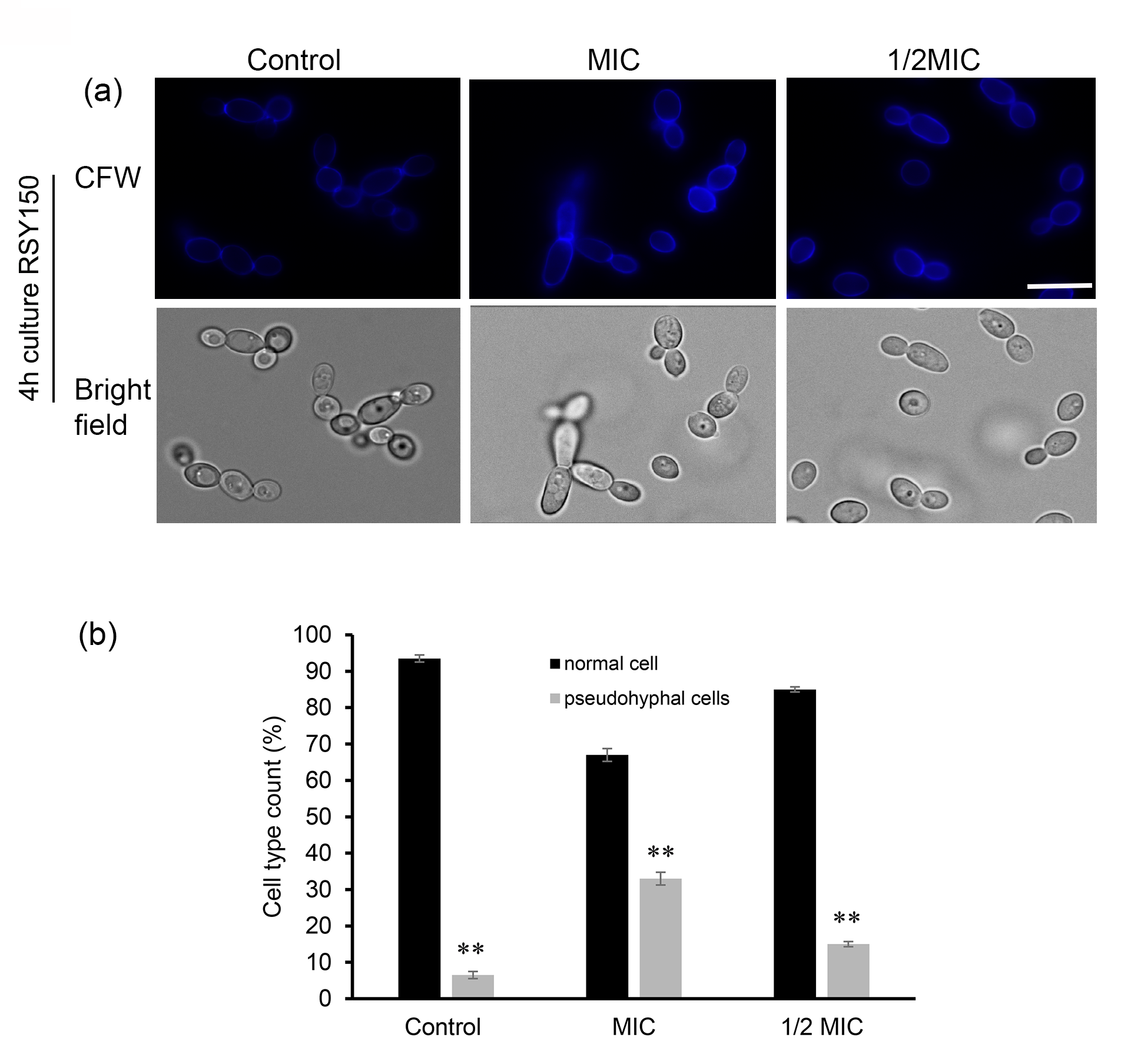

Supplement: Supplementary file 3 — Additional file 3: Figure S2. (a) Cell cycle stress induced pseudohyphae in CNB oil exposed RSY150. Mid log phase RSY150 after 4 h exposure to CNB oil at MIC and 1/2 MIC, were stained with CFW. Images are epifluorescence (top panel) and bright field (BF; bottom panel). Bar = 5 μm. (b) Quantification of cells in control, MIC and 1/2 MIC from (a). Double asterisks represent p < 0.05. [file 40694_2018_46_MOESM3_ESM.tif]

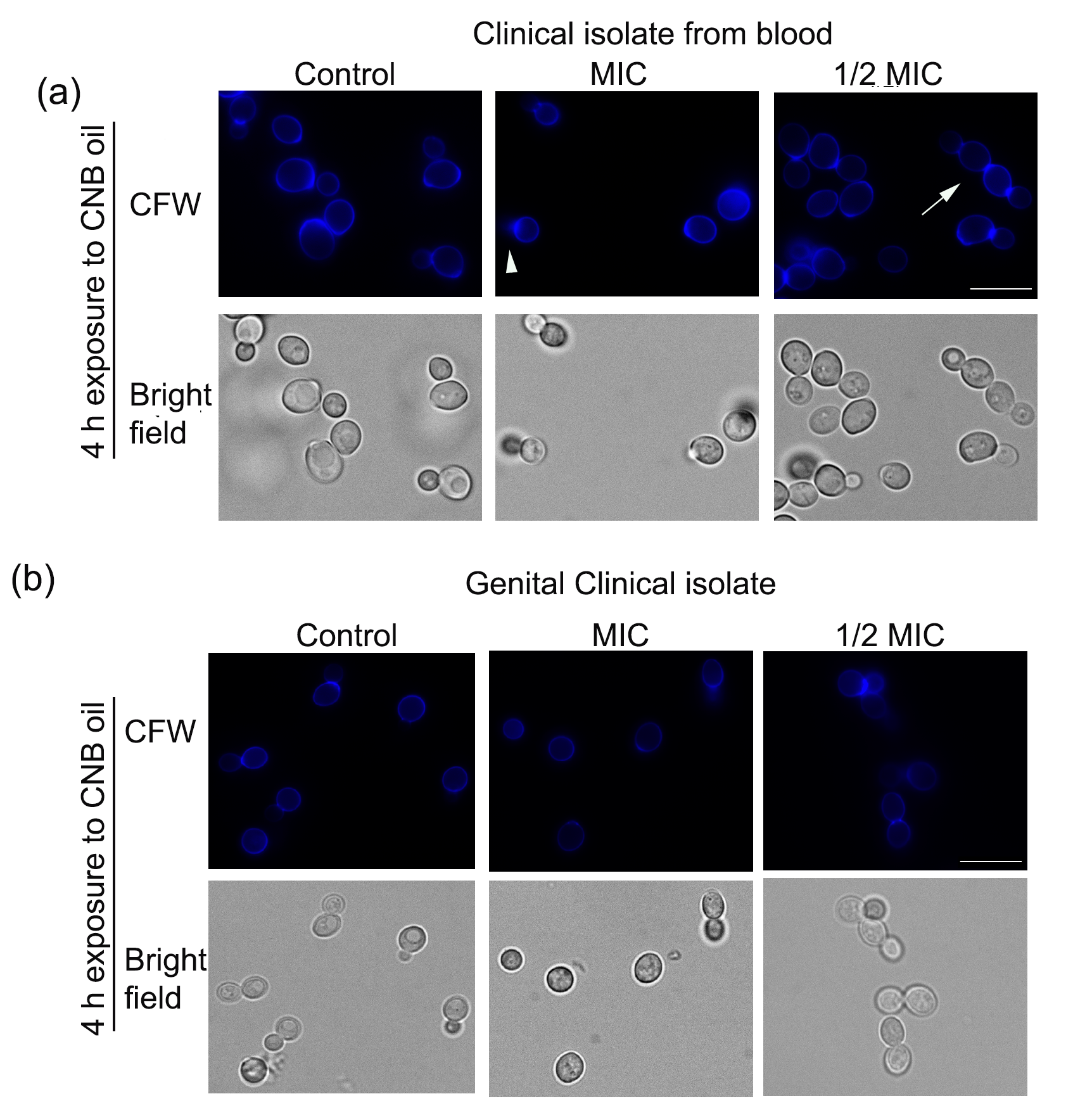

Supplement: Supplementary file 4 — Additional file 4: Figure S3.(a) Clinical isolate exposed to CNB oil showed increased chitin content. The clinical isolate from blood at log phase after 4 h exposure to CNB oil at MIC and 1/2 MIC were stained with CFW. Images represent CFW (top panel) and bright field (BF; bottom panel). Bar = 5 μm. (b) Genital clinical isolate with comparable MIC to RSY150 showed a normal chitin distribution. [file 40694_2018_46_MOESM4_ESM.tif]

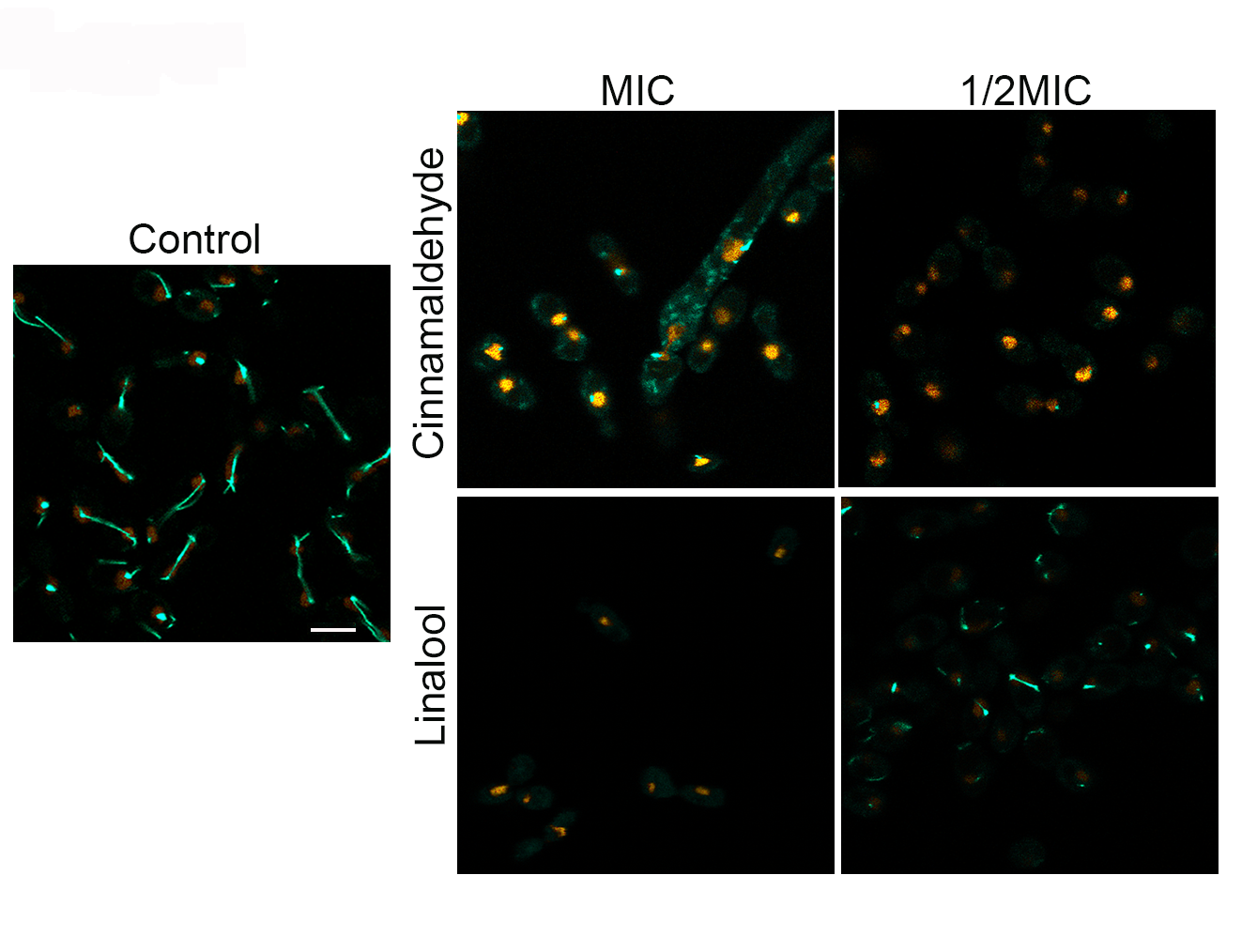

Supplement: Supplementary file 5 — Additional file 5: Figure S4. Spindle morphology of cinnamaldehyde and linalool treated C. albicans. Live LSCM of mid log phase cells after 4 h exposure to MIC and 1/2 MIC of cinnamaldehyde and linolool. Cinnamaldehyde treated C. albicans at MIC showed a similar spindle morphology of those treated with CNB oil at MIC, whereas linalool treated cells showed a complete absence of tubulin at MIC, with decreased cell size. At 1/2 MIC for both cinnamaldehyde and linalool, tubulin expression appeared as fluorescent spots near the nucleus. Bar = 5 μm. [file 40694_2018_46_MOESM5_ESM.tif]
